# Multi-disciplinary support of infant feeding choice for parents living with HIV: experience across a guideline change

**DOI:** 10.3389/frph.2026.1867965

**Published:** 2026-06-26

**Authors:** S. Gogia, A. H. Latham, J. L. Gerard, M. Moore, A. Bailey, K. Momin, E. Barba Gutierrez, S. Gillespie, G. Mirani, M. E. Paul, C. Wallace, J. R. McKinney

**Affiliations:** 1 Division of Maternal Fetal Medicine, Department of Obstetrics & Gynecology, Baylor College of Medicine, Houston, TX, United States; 2 Department of Obstetrics & Gynecology, Baylor College of Medicine, Houston, TX, United States; 3 School of Medicine, Baylor College of Medicine, Houston, TX, United States; 4 Division of Immunology, Allergy, and Retrovirology, Department of Pediatrics, Baylor College of Medicine, Houston, TX, United States; 5 Harris Health System, Houston, TX, United States

**Keywords:** breastfeeding, HIV, infant feeding, multidisciplinary support, postpartum, pregnancy

## Abstract

**Introduction:**

Support for infant feeding choice for people living with HIV (PLWH) in the United States has changed, with national guidelines encouraging collaborative decision making to support infant feeding decisions in 2023. In 2020, our institution developed a multi-disciplinary approach supporting patient-centered infant feeding counseling. Our study's objective was to describe the outcomes and experiences of parents engaging with this model.

**Methods:**

Electronic records were reviewed for PLWH initiating Obstetric care between January 2020 and September 2025 in a maternal HIV program in Houston, Texas. Information regarding demographics, HIV care, participation in infant feeding support model, and feeding experiences was collected. Data was summarized using descriptive statistics.

**Results:**

336 PLWH initiated care during the study period. 75 considered breastfeeding, 51 completed Pediatric Retrovirology consultation to discuss benefits/risks, and 37 breastfed. No HIV transmissions occurred with breastfeeding. Of the 37 parents who breastfed, 92% maintained undetectable viral loads throughout breastfeeding. Median duration of breastfeeding was 47 days. 69% of these parents reported challenges with breastfeeding contributing to premature weaning. Post-delivery retention in HIV care was a significant challenge for parents (only 38% attended two of their own HIV care visits within 1 year), compared to neonates attending 84%–92% of Pediatric Retrovirology appointments for routine postnatal monitoring.

**Discussion:**

Our institution observed increasing interest in breastfeeding for PLWH, especially since the update of US guidelines. Half of our cohort who expressed interest ultimately breastfeed, but significant challenges remain limiting duration of breastfeeding and retention in postpartum HIV care.

## Introduction

Support for people living with HIV (PLWH) in the United States (US) has changed over time. Historically, breastfeeding was strongly discouraged in the US due to a significant postnatal HIV transmission risk in the absence of antiretroviral therapy (ART), with replacement feeding with formula or pasteurized donor milk removing this risk (1). However, advances in ART have dramatically reduced this risk. Contemporary data, while limited, suggests that with sustained maternal viral suppression and infant prophylaxis, the risk of transmission during breastfeeding is 0.1% per month or less (2–5).

Alongside clinical advances, there is increasing recognition of the complex factors that influence infant feeding decisions among PLWH. Qualitative studies demonstrate that decisions are shaped not only by transmission risk, but also social, cultural, and structural factors (6–8). For many parents, infant feeding decisions include consideration of known maternal and infant health benefits as well as parental identity, bonding, and cultural expectations (6, 7). These pressures may be intensified for individuals who have not disclosed their HIV diagnosis, as formula feeding can risk inadvertent disclosure and exposure to stigma or interpersonal conflict (6–8). In this context, parents must balance psychosocial safety, autonomy, and identity, with the tension between public health recommendations and cultural norms.

Evolving transmission risk data and recognition of these socio-cultural complexities prompted the January 2023 revision of the Department of Health and Human services Perinatal HIV Guidelines to support patient-centered shared decision-making about infant feeding (9). However, implementation in the US remains inconsistent across clinical settings. Studies continue to demonstrate variability in provider knowledge, comfort, and counseling practices surrounding infant feeding for PLWH (10).

Multidisciplinary models, which can help build a supportive environment around the parent, have emerged as a promising strategy to support PLWH in making infant feeding choices. Although models differ by site, most reinforce the importance of early counseling, support for adherence to ART to ensure continued viral suppression, infant prophylaxis, lactation support, and close postpartum monitoring for parents who chose to breastfeed (3, 11). While data suggests these approaches can minimize transmission risk, real-world implementation data is limited, particularly in US settings navigating evolving guidelines (3, 5, 10, 11).

Our institution serves a diverse population of PLWH from varied religious, cultural, and socioeconomic backgrounds, and hearing patient experiences prompted early recognition of the complex factors influencing infant feeding decisions. Many of our patients expressed an interest in, or intention to, breastfeed despite US guidelines prior to 2023 recommending replacement feeding for PLWH. In an effort to safely support these patients and provide informed care, our large, public hospital-affiliated perinatal HIV clinic in Houston, TX implemented a multidisciplinary, patient-centered infant feeding counseling model prior to the 2023 guideline changes. This model includes consultation and support from high risk Obstetric and Pediatric HIV providers as well as lactation consultants.

To address the literature gap regarding PLWH and infant feeding, we conducted a retrospective cohort study of PLWH receiving prenatal care at our institution. This study aims to characterize patient interest in breastfeeding, engagement with counseling and HIV care, and infant feeding outcomes within this model. Please note that in this manuscript, “breastfeeding” is used to describe feeding a child the parent's own milk (directly or expressed). We recognize alternative terminology, such as “chestfeeding,” may be preferred by some individuals. Gender-inclusive language is used throughout, except when reflecting terminology from cited sources.

## Methods

This retrospective cohort study reviewed all pregnant PLWH who initiated Obstetric care between January 1, 2020 and September 30, 2025 with the maternal HIV program at Harris Health, a large county healthcare system that includes the Houston, TX metropolitan area.

The program instituted a multi-disciplinary infant feeding care model in 2020 including counseling on infant feeding choice at initiation of prenatal care for all patients. Any person who is considering breastfeeding is then offered consultation with our institution's Pediatric Retrovirology team to further discuss benefits and risks. Parents who choose to breastfeed are offered lactation support during hospital admission and postpartum. They are followed closely by our Obstetric and HIV care teams postpartum and monthly viral loads are obtained while they are breastfeeding. Infants are followed by the Pediatric Retrovirology team and undergo HIV testing at regular intervals through weaning and then up to 18 months of age.

Pregnant PLWH were included in the study if they were 18 years of age or older, received prenatal care within our clinic, considered their own breastmilk for infant feeding, and delivered a liveborn neonate or had a loss after 14 weeks gestation during the study time period. Patients who experienced a loss after 14 weeks were included because breastfeeding interest was documented starting at their first prenatal visit. Patients who experienced a loss were supported with our institution's standard bereavement and lactation protocols that are not specific to our multidisciplinary model. All pregnancies that met criteria for each individual were included in analyses as independent events due to the importance of describing experience with the infant feeding counseling model with each pregnancy.

The electronic medical record was reviewed and data was collected for the following categories: demographics, Obstetric care (pregnancy, delivery, and postpartum factors), HIV-related care (HIV care visits, laboratory data, antiretroviral therapy), neonatal care (neonatal ICU admission, testing results, Pediatric Retrovirology surveillance visits), and feeding details (feeding methods, duration, and challenges). We approximated social vulnerability using the CDC and Agency for Toxic Substances and Disease Registry Social Vulnerability Index (12). Viral suppression was defined as HIV-1 RNA (viral load) copies <50 copies/mL. Neonatal and postpartum data were collected through the last documented encounter prior to data collection in February 2026. Outside records, when available, were used to confirm HIV primary care follow-up. Data was coded and entered into a secure electronic REDCap database (13, 14).

Summary statistics were used to describe categorical variables, and chi-square tests or Fisher's exact tests were used for testing significance. Continuous variables were described as means with standard deviation (SD) if normally distributed, or medians with interquartile ranges (IQRs) for non-normally distributed data. Wilcoxon Rank Sum tests were used for testing for significant differences between groups for continuous data. No imputation was made for missing data. All analysis were performed using R studio software (15).

The Baylor College of Medicine institutional review board (IRB) approved this study and waived informed consent (Protocol H-18412).

## Results

**Figure 1:**
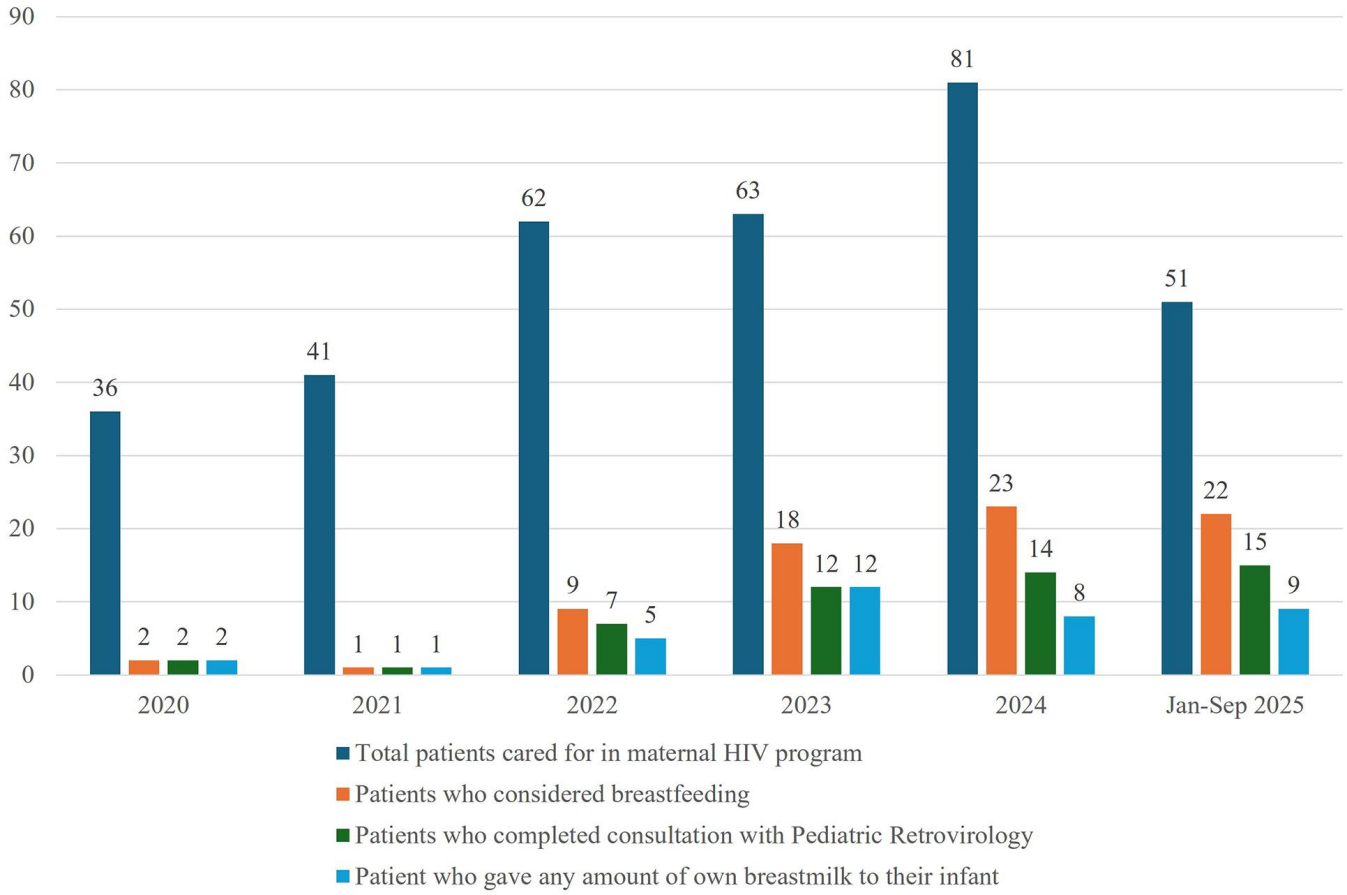
Parent engagement in multidisciplinary infant feeding counseling model.

A total of 336 PLWH received care in the maternal HIV program during the study period. Eightof these represented repeat pregnancies for patients who had received care with our team in a prior pregnancy. Seventy-five people (22%) expressed interest in breastfeeding and were referred for consultation with Pediatric Retrovirology. All 75 of these patients went on to have a live birth. Fifty-one of those referred (68%, 15% of all patients) completed consultation with our Pediatric Retrovirology team at a mean gestational age of 32.6 weeks (SD 3.9). Following consultation, 43 patients (84%, 13% of all patients) expressed intent to breastfeed on arrival for delivery admission, and ultimately 37 of these 43 (86%, 11% of all patients) gave breast milk to their infant (Figure 1). Over the 5-year study period, patient consideration of breastfeeding, or potentially recognition of patient consideration of breastfeeding increased (2020: 6%, January–September 2025: 43%). The number of patients who fed their own breastmilk to their infants also increased (2020: 6%, January–September 2025: 18%) (Figure 1).

With the goal of examining our counseling and support process, the remainder of the results will concentrate on people who considered breastfeeding (*n* = 75). The median age of this cohort was 31 years old. Significant differences were noted between individuals that fed their own breastmilk to their infant and those that replacement fed only with regards to race/ethnicity and primary language, with increased rates of breastfeeding among patients who were Black non-Hispanic (*p* = .0006) and who primarily spoke English (*p* = 0.035). Social vulnerability was high overall in this cohort [overall SVI median (IQR) 0.82 (0.47–0.91)], with increased housing and transportation vulnerability observed in those that replacement fed only [SVI Theme 4 median (IQR) 0.51 (0.28–0.76) vs. 0.71 (0.48–0.88), *p* = 0.033]. No differences were observed in other baseline characteristics. Roughly half were born in the US (56%) and in a long term partnership (50%). Most patients had public insurance (87%). 23% had a history of substance use disorder, and 24% were taking medication for a mental health disorder during their pregnancy.

In general, individuals were well engaged in prenatal care, with the majority (67%) initiating prenatal care in the first trimester at median gestational age at entry to care of 11 weeks (IQR 9–16 weeks). Patients attended an appropriate number of prenatal visits, with median number of prenatal visits 11 (IQR 8–13) for the total cohort (10 for patients who breastfed vs. 11 for patients who did not, *p* = 0.635). Most (89%) patients ultimately delivered at term, and the majority (56%) had a vaginal delivery. Cesarean deliveries were performed for routine Obstetric indications, with no patients in this cohort undergoing a Cesarean delivery for elevated viral load. There were 12 (16%) repeat pregnancies within 2 years of delivery (Table 1).

**Table 1:** Demographic, pregnancy, postpartum, and neonatal characteristics by infant feeding choice.

Characteristic	Considered breastfeeding	Fed own breastmilk to infant	Replacement feeding only	*p*
	*N* = 75	*N* = 37	*N* = 38	
Age, years, median (IQR)	31 (27–34)	30 (27–34)	31 (26–35)	0.983
Country of birth				0.568
US	42 (56)	18 (48.7)	22 (57.9)	
Outside of US^a^	33 (44)	19 (51.4)	16 (42.1)	
Race/Ethnicity				**0.006**
Asian	1 (1)	1 (2.7)	0 (0)	
Black Hispanic	4 (5)	2 (5.4)	2 (5.3)	
Black Non-Hispanic	50 (67)	30 (81.1)	20 (52.6)	
White Hispanic	15 (20)	1 (2.7)	14 (36.8)	
White Non-Hispanic	5 (7)	3 (8.1)	2 (5.3)	
Primary language				**0.035**
English	55 (73)	32 (86.5)	23 (60.5)	
Spanish	14 (19)	3 (8.1)	11 (28.9)	
Other	6 (8)	2 (5.4)	4 (10.5)	
Insurance at delivery				1.000
Government funded	65 (87)	32 (86.5)	33 (86.8)	
Commercial	10 (13)	5 (13.5)	5 (13.2)	
Marital status				0.307
Married	25 (33)	14 (37.8)	11 (28.9)	
Single	37 (49)	15 (40.5)	22 (57.9)	
Long term partnered	13 (17)	8 (21.6)	5 (13.2)	
History of substance use	17 (23)	8 (22)	9 (24)	1.000
History of mental health disorder	30 (40)	14 (38)	16 (42)	0.888
Mental health disorder treated during current pregnancy	18 (24)	7 (19)	11 (29)	0.456
EPDS				
First trimester, ≥10	18 (27)	8 (26)	10 (29)	0.963
Third trimester, ≥10	11 (19)	8 (27)	4 (12)	0.250
Postpartum, ≥10	11 (18)	5 (15)	7 (21)	0.879
Overall SVI, median (IQR)	0.820 (0.427–0.909)	0.580 (0.335–0.900)	0.843 (0.620–0.910)	0.148
SVI Theme 1: Socioeconomic status	0.818 (0.470–0.938)	0.780 (0.350–0.943)	0.846 (0.760–0.936)	0.167
SVI Theme 2: Household characteristics	0.501 (0.201–0.854)	0.414 (0.129–0.773)	0.556 (0.265–0.856)	0.494
SVI Theme 3: Racial/ethnic minority status	0.860 (0.777–0.925)	0.850 (0.716–0.928)	0.863 (0.806–0.918)	0.771
SVI Theme 4: Housing type and transportation	0.627 (0.325–0.870)	0.509 (0.283–0.759)	0.710 (0.481–0.884)	**0.03**
Nulliparous	16 (21)	11 (29.7)	5 (13.2)	0.14
Entry to prenatal care				0.37
First trimester	50 (67)	25 (68)	25 (66)	
Second trimester	20 (27)	11 (30)	9 (24)	
Third trimester	5 (7)	1 (3)	4 (10)	
Gestational age at delivery				1.000
Preterm <37 weeks	8 (11)	4 (11)	4 (10)	
Term ≥37 weeks	67 (89)	33 (89)	34 (90)	
Mode of delivery				0.230
Vaginal	42 (56)	18 (49)	24 (63)	
Cesarean^b^	33 (44)	19 (51)	14 (37)	
Attended ≥1 Obstetric postpartum visit	72 (96)	36 (97)	36 (95)	1.000
Repeat pregnancy <2 years postpartum^c^	12 (16)	8 (22)	4 (10)	-
NICU admission	21 (28)	10 (27)	11 (29)	0.941
Length of stay in NICU, days, median (IQR)	5 (3–10)			

Data are presented as *n* (%) unless otherwise indicated. Intermittent item missingness leads to some column sums adding up to less than total.

a Birth country in Africa (*n* = 18), Central or South America (*n* = 11), Asia (*n* = 1).

b Cesarean for routine Obstetric indications only, no persons required Cesarean for elevated viral load in this cohort.

c Many participants have not yet reached 2 years after delivery (*n* = 37).

The bold values indicate variables with significant differences between groups.

Most individuals (75%) were diagnosed with HIV prior to pregnancy, with 9 (12%) having acquired HIV perinatally and 47 (63%) acquiring HIV after birth but before pregnancy. Eleven and eight patients were newly diagnosed in the first and second trimesters of pregnancy respectively through routine prenatal testing. For patients diagnosed with HIV prior to pregnancy (*n* = 56), 48% had seen their HIV care provider in the last 6 months. Slightly more of this group of patients elected to breastfeed (63%) rather than replacement feed (31%), though this difference did not reach statistical significance (*p* = .071). Seventy-nine percent of patients diagnosed with HIV prior to pregnancy had an undetectable viral load upon entry to prenatal care. All patients were maintained on ART throughout their pregnancies. Almost all patients, including those diagnosed prior to and during pregnancy, had an undetectable viral load at delivery, (97%), and only 27% received intravenous zidovudine during labor. People who gave breastmilk to their infants maintained undetectable viral loads in 92% of cases. Of the three patients who did not, viral loads were 51, 53, and 60 copies/mL. The patient with a viral load of 51 copies/mL was retested and found to be undetectable, so continued breastfeeding, while the other two patients elected to wean their infants after counseling. Only 31% of the patients were optimally retained in care (had completed two HIV care visits) by 12 months postpartum (Table 2).

**Table 2:** Maternal and neonatal HIV care characteristics by infant feeding choice.

Characteristic	Considered breastfeeding	Fed own breastmilk to infant	Replacement feeding only	*p*
	*N* = 75	*N* = 37	*N* = 38	
Timing of HIV diagnosis				0.377
Pre-existing diagnosis: perinatal acquisition	9 (12)	6 (16)	3 (8)	
Pre-existing diagnosis: horizontal acquisition	47 (62)	24 (65)	23 (60)	
New diagnosis in first trimester	11 (15)	5 (14)	6 (16)	
New diagnosis during second trimester	8 (11)	2 (5)	6 (16)	
HIV status disclosed to partner	58 (77)	28 (76)	30 (79)	1.000
On ART prior to pregnancy^a^	49 (88)	27 (90)	22 (85)	0.191
Last HIV care visit prior to prenatal care^a^				0.071
<6 months	27 (48)	19 (63)	8 (31)	
6–12 months	8 (14)	2 (7)	6 (23)	
12–24 months	10 (18)	6 (20)	4 (15)	
Undetectable viral load (<50 copies/mL)				
First prenatal visit	45 (60)	26 (70)	19 (50)	0.120
Throughout third trimester	67 (89)	35 (95)	32 (84)	0.280
At delivery	73 (97)	36 (97)	37 (97)	1.000
During breastfeeding		34 (92)		–
ARV regimen at delivery				0.093
BIC/FTC/TAF	43 (57)	16 (43)	27 (71)	
DTG + FTC/TDF or FTC/TAF	12 (16)	8 (22)	4 (11)	
Received AZT during delivery	20 (27)	7 (19)	13 (34)	0.216
Postpartum RIC *(≥2 HIV care visits within first 12 months postpartum)*	23 (31)	14 (38)	9 (24)	0.338
1 HIV care visit in first year postpartum	21 (28)	10 (27)	11 (29)	
No documented HIV care visit in first year postpartum^b^	31 (41)	13 (35)	18 (47)	
Attendance at pediatric retrovirology visits				
2 weeks	66 (88)	32 (87)	34 (90)	0.227
2 months	61 (81)	34 (92)	27 (71)	0.158
4 months	59 (79)	31 (84)	28 (74)	0.673
18 months^c^	16 (21)	10 (27)	6 (16)	–
Neonatal HIV testing negative at 2 weeks, 2 months, and 4 months	75 (100)	37 (100)	38 (100)	1.000

Data are presented as *n* (%) unless otherwise indicated. Intermittent item missingness leads to some column sums adding up to less than total.

a For pre-existing diagnoses only: Considered breastfeeding *n* = 56, Fed own breastmilk to infant *n* = 30, Replacement feeding only *n* = 26.

b Patients may have obtained care outside our system or outside nstitutions participating in Care Everywhere, a network through which outside records are available.

c Many participants have not yet reached 18 months of life for the child.

Summary of breastfeeding experience for this cohort is found in Table 3. Many (40%) of patients reported breastfeeding following a prior pregnancy. Interestingly, a majority of those parents (77%) reported previously breastfeeding in the United States. Ultimately, 37 of 75 (49%) who initially expressed interest in breastfeeding gave breast milk to their infant. Six expressed intent to breastfeed at time of delivery admission but elected to formula feed after delivery. Three of those parents delivered at a hospital outside of our system and were counseled to formula feed, one obtained a new job with a start date of 1 month postpartum and decided to formula feed for ease of transition, one had a viral load that was detectable at time of delivery despite previously having an undetectable viral load, and one patient's partner was concerned about risk of transmission after inpatient counseling so the couple ultimately decided not to breastfeed. Parents ultimately breastfed for a median of 47 (IQR 8–121) days, ranging from 1 to 415 days. One person is still breastfeeding to date, and thus total duration is unknown. Of the 36 patients with known duration of breastfeeding, only 20 (55%) breastfed for longer than 30 days and 10 (28%) breastfed for less than or equal to 7 days. All infants received prophylaxis per our institution's protocols: zidovudine for 2 weeks and nevirapine through 4 weeks after last breast milk exposure (Table 3).

**Table 3:** Breastfeeding history and experience.

Characteristic	Considered breastfeeding	Fed own breastmilk to the infant
	*N* = 75	*N* = 37
Breastfed in a prior pregnancy	30 (40)	
Previously breastfed in US	20 (27)	
Previously breastfed outside the US^a^	8 (11)	
Intended to breastfeed at delivery admission	43 (57)	
Re-counseled by inpatient pediatric team regarding feeding choice	34 (92)	
Fed own breastmilk to infant	37 (49)	
Lactation consult during delivery admission		33 (89)
Number of times seen by lactation, median (IQR)		2 (1–3)
Duration of breastfeeding, days, median (IQR)^b^		47 (8–121)
0–7 days		10 (27)
8–14 days		3 (8)
15–30 days		3 (8)
31–60 days		5 (14)
61–90 days		3 (8)
91–120 days		3 (8)
121–150 days		3 (8)
151–180 days		2 (5)
>180 days		4 (11)
Challenges with breastfeeding reported to clinician		27 (69)
Low supply		18 (67)
Fatigue		2 (7)
Mastitis		2 (7)
Pain with feeding		5 (19)
Poor latch		10 (37)
Nipple trauma		2 (7)
Lack of support from partner, family or friends		1 (4)
Issues with pump		2 (7)
Separation from infant		1 (4)
Anxiety about transmission		2 (7)
Supplemented with formula		13 (35)
Infant DOL 1–2		8 (62)
Infant DOL ≥3		5 (38)

Data are presented as *n* (%) unless otherwise indicated. Intermittent item missingness leads to some column sums adding up to less than total.

a Countries included Mozambique (2), one each from Nigeria, Democratic Republic of Congo, Burundi, Equatorial Guinea, Honduras, Colombia.

b One patient had not yet stopped breastfeeding at time of data collection.

Seventy-three percent of patients who breastfed reported challenges that affected their breastfeeding journey, most commonly low supply (67%) and poor latch (37%). Other challenges cited included fatigue, issues with electric breast pump, mastitis, pain with feeding, nipple trauma, lack of support from loved ones, separation from infant (for example due to NICU admission), and anxiety about HIV transmission. Thirteen (35%) of patients who breastfed supplemented with formula, 8 within the first 1–2 days of life. Ultimately, 16 parents (46%) reported weaning their infants from breast milk due to low supply (Table 3).

No lactational transmissions of HIV occurred in this cohort of breastfeeding dyads at the time of this publication. Adherence with neonatal Pediatric Retrovirology follow up was excellent, ranging from 88% attendance at the 2 week visit to 79% at the 4 month follow up. Only 16 (21%) have thus far attended the 18-month follow up, as many breastfed infants have not yet reached this age (Table 2).

## Discussion

Our patients' experiences and interest in a full range of infant feeding options pushed our clinical program to develop the multidisciplinary infant feeding care model described in this report in 2020, before US national guidelines were updated in 2023 (16). We have observed exponentially increasing interest, or perhaps increased recognition of our patients' interest in breastfeeding, since the updated guidelines were published and over the 5 years that our program has been in effect. Although the number of patients considering and actually giving breastmilk to their infants has increased over time with the support provided by our program, disparities persist based on race/ethnicity, primary language, social vulnerability such as housing and transportation resources, and differences in support based on delivery hospital. The majority of patients who breastfed identified as Black Non-Hispanic, and many had breastfed in a previous pregnancy which indicate that our findings may reflect cultural differences, fear of disclosure of HIV status, or having previously breastfed in a different country. While qualified interpreters are consistently used in the outpatient setting for counseling, differences in primary language may have limited patient engagement with our primary model or resulted in conflicting information being provided if qualified interpreters were not used in the inpatient or postpartum setting. Lastly, housing and transportation resources may limit ability to present to clinic to address challenges with breastfeeding such as low supply and poor latch and also may be indicative of broader social stressors such as a need to return to work shortly after delivery. Ultimately, our data is limited in its ability to determine whether these decisions are reflective of differences in patients' preferences regarding infant feeding, or if they represent barriers to breastfeeding in these populations. While other qualitative studies have demonstrated cultural differences between patients that are in line with our data on race/ethnicity and decision to breastfeed, housing and transportation resources and primary language have not yet been identified as significant factors in the decision to breastfeed for North American cohorts of PLWH (5, 7, 8). This data also highlights, on a small scale, the differences in peripartum support for infant feeding across varying hospital systems in the US, and how important the attitude/support is at the delivery hospital itself. Standardized approaches will be needed to improve access to equitable support for PLWH in infant feeding choice regardless of where they live.

The challenges that our patients who chose to breastfeed faced are more broadly reported in the literature than the above disparities, particularly including duration of breastfeeding and low supply, though these challenges were observed in a higher percentage of our cohort than others (5, 8). Duration of breastfeeding in our cohort was limited to a median time of only 47 days. While adherence to HIV treatment and viral suppression played a role in weaning for two patients in our cohort, the majority of patients reported premature weaning due to low supply or poor latch. It is possible that challenges were underreported, as we were limited to issues self-reported by parents during postpartum, pediatric, and lactation visit notes as well as telephone encounters. Whether or not low supply is a specific challenge for PLWH or if decisions to wean were multifactorial also could not be ascertained from this retrospective work and will require further examination of larger and more diverse cohorts as well as qualitative studies. Challenges with supply were further reflected in the need for formula supplementation by many patients in our breastfeeding cohort, with 22% supplementing during the first two days of their infant's life. Evidence and expert opinion regarding the importance of avoiding formula varies, with older studies suggesting an increased risk of transmission with mixed feeding, but scant data from the era of contemporary ART, or within multi-disciplinary support models such as our institution's (17–20). An update to US guidelines in 2024 commenting on risk reduction of transmission recommended exclusive breastfeeding for the first 6 months of life, while acknowledging that there may be scenarios where formula supplementation is needed, commenting specifically that there is no evidence that formula supplementation increases the risk of HIV acquisition in the breastfed infant in the context of parental ART and viral suppression (21, 22).While our study is underpowered to comment specifically on the question of mixed feeding, it provides unique data regarding mixed feeding that, to our knowledge, has not been otherwise published in US cohorts. Despite one-fifth of our breastfeeding cohort supplementing with formula, zero cases of transmission were seen.

Finally, in contrast to this cohort's excellent adherence with neonatal follow up, postpartum retention in HIV care was poor. Only 38% of breastfeeding parents had two visits documented with their HIV PCP within the first year postpartum. This figure consistent with our institution's general rate of postpartum retention in care, as well as several other studies conducted in varying settings across the US, though it is possible that our patients obtained HIV care outside our system or network of available outside medical records (19, 23–26). Though parents maintained excellent viral suppression throughout the duration of breastfeeding, the loss to HIV care follow up demonstrates significant vulnerability in the first year following delivery. Retention in care will likely play an even bigger role in ensuring reduction of transmission risk with breastfeeding as systems evolve to better support PLWH in their decision to breastfeed, as well as support longer durations. Concerted efforts are necessary to improve understanding of why these lapses in care occur, as continued retention in care and viral suppression are vital not only for long term individual health outcomes for PLWH, but for their families as well.

The primary strength of our study is that it represents, to our knowledge, the largest single site cohort of PLWH in North America who opted to breastfeed, spanning a time period from before to after the national guideline changes in 2023. Our institution's proactive approach in creating a multi-disciplinary model demonstrates the importance and efficacy of coordinated care in supporting PLWH with their infant feeding goals.

We recognize that collaborative care amongst the large array of specialists comprising our care model will not be possible in many settings, limiting the generalizability of this data and approach. Other limitations lie in the descriptive nature of the study and relatively small sample size. Further, the retrospective nature of this study requires that it relies on information obtained from record review and thus is subject to the accuracy and detail of information that is recorded in the electronic medical record. Despite these limitations, the detailed data collected will help inform future larger scale multi-site and national studies as similar multi-disciplinary models and approaches are adopted across the United States.

To address the challenges inherent to a retrospective study, future qualitative work is planned with our cohort to better understand the experiences of PLWH interacting with our multidisciplinary infant feeding support model with the goal of improving the program to better support parents in reaching their infant feeding goals.

Infant feeding choice is a deeply personal decision that should be adequately and equitably supported for all PLWH no matter the setting or geographic location in which they receive care. Experience with our care model over the last 5 years has shown improvement in support for infant feeding choice but also highlights significant barriers that remain for so many of our patients. These barriers must be addressed within our own system and across the US in order to reduce bias, promote justice, and support healthy families for all persons living with HIV.

## Data Availability

The raw data supporting the conclusions of this article will be made available by the authors, without undue reservation.
